# Supplementary figures and images for: Unveiling inter-embryo variability in spindle length over time: Towards quantitative phenotype analysis
Source: PLoS Comput Biol. 2024 Sep 5;20(9):e1012330. doi: 10.1371/journal.pcbi.1012330 (PMC11376571; doi:10.1371/journal.pcbi.1012330)

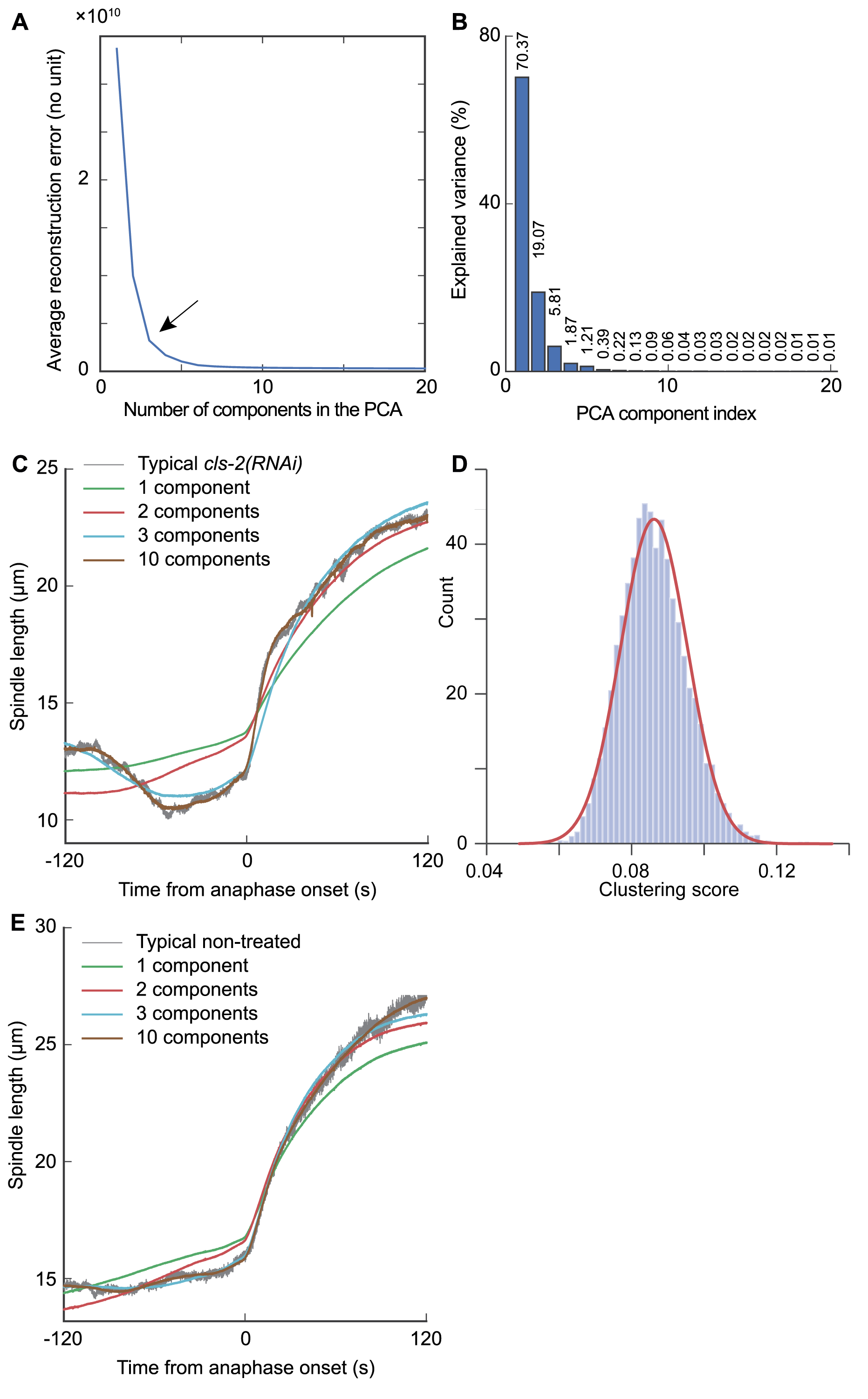

Supplement: S1 Fig — (A) Decrease of the L2 error, i.e. the sum of the squares of the residuals, when increasing the number of dimensions in the PCA method. We suggest that 3 dimensions are optimal as it corresponds to the corner of the L-shaped curve (arrowhead). (B) Percentage of explained variance by each PCA component. (C, E) (grey) Comparing the raw spindle elongation of an exemplar single embryo labelled by GFP::ɣTUB, treated by (C) cls-2(RNAi) during 24 h or (E) non treated, and imaged at 18°C. (coloured curves) We reconstructed the variability around the average with 1–3 and 10 PCA components and added the average elongation of all conditions used in this paper. The third component (archetype) was essential to recapitulate the key features of the experimental trajectories, especially the transient spindle limited-elongation / shortening before anaphase onset. (D) Histogram of clustering scores from a PCA with scrambled labels (§1 in S1 Methods) to be compared to PCA with real labels scoring 1.86. The red line depicts the maximum likelihood Gaussian fit. (TIF) [file pcbi.1012330.s001.tif]

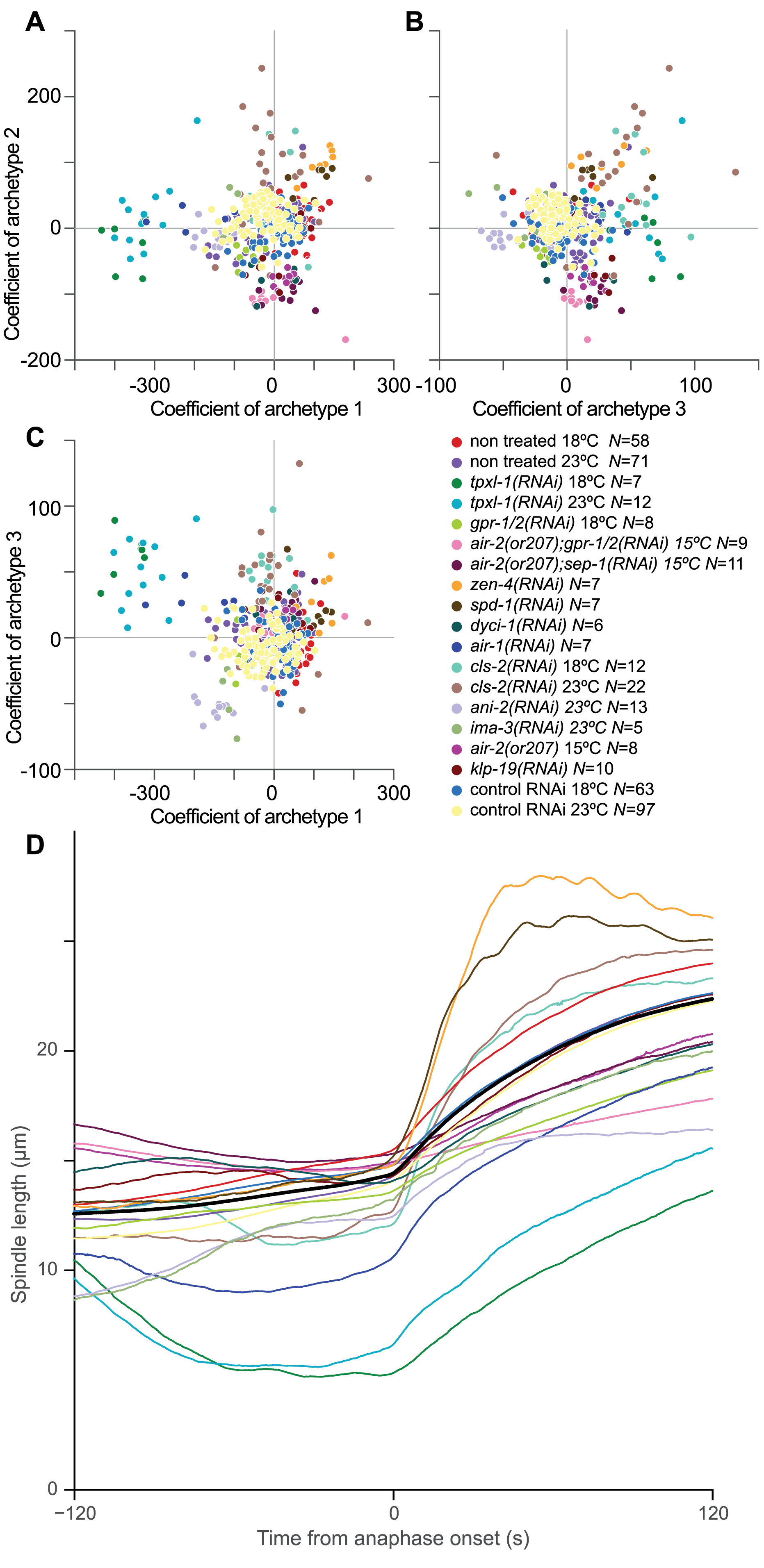

Supplement: S2 Fig — Green thin lines report individual embryo elongation curves for the N = 5 embryos with lowest coefficient 3, C3-¯≃-21.4±4.3. We first selected embryos with coefficient 2 between the first and third quartiles, termed mid-coefficient 2 embryos (N = 28). Then among these, we took 15% of the embryos with extreme coefficient 3. Doing so, coefficient 3 of the extreme pool is clearly different compared to the one of mid-coefficient 2 embryos, C3¯≃-5.55±2.05. In contrast, the coefficients 1 and 2 are similar in the two groups; they read C1-¯≃53.9±16.9 and C2-¯≃6.8±3.7 for the group with lowest coefficient 3 compared to C1¯≃47.2±5.02 and C2¯≃9.73±1.23 for mid-coefficient 2 group. The thick coloured line corresponds to the averages over these groups. The thicker blue line corresponds to the average over mid-coefficient 2 embryos. Compared to this latter average, a faster spindle elongation in late metaphase is visible in the low-coefficient-3 embryos average. All experiments were done using strain TH27, acquired at 18°C. Individual embryo and averaged tracks were smoothed using a 1.5 s-running-window median. (TIF) [file pcbi.1012330.s002.tif]

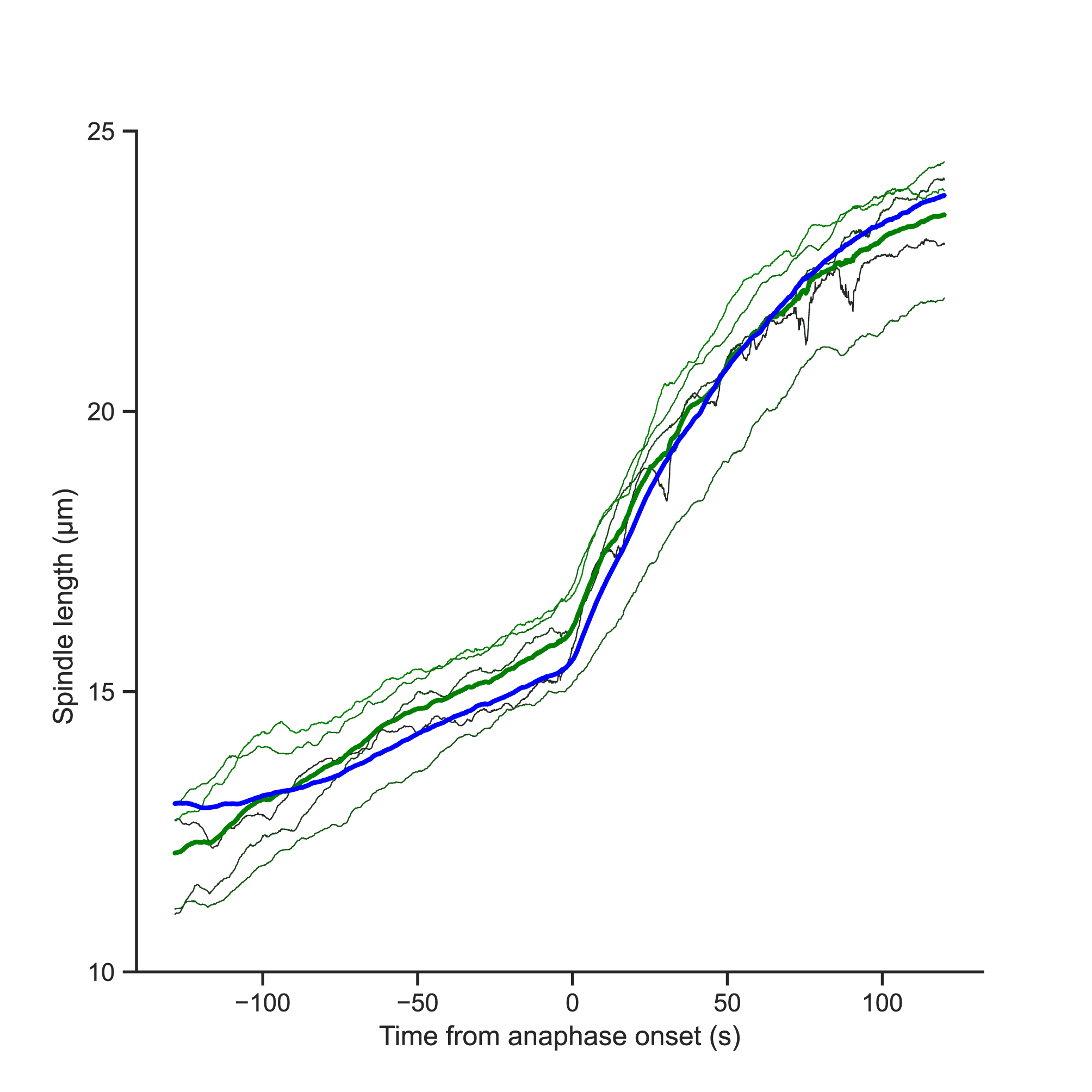

Supplement: S3 Fig — Imaging was performed at 18°C except otherwise stated. (A) Coefficients corresponding to the first two main archetypes (PCA components). (B) Similar plots for the second and third archetypes, and (C) for the first and third archetypes. Colours refer to genetic perturbations. Grey lines depict the 0 on each axis. All experiments were done using strain TH27 except the ones featuring air-2(or207), which used JEP31. An interactive 3D plot is attached as S2 File. (D) Pole-pole distance (spindle length) averaged per condition and plotted during metaphase and anaphase for the cases displayed in panels A-C. Multiple conditions treating the same gene by RNAi or mutating it are merged. Averaged tracks were smoothed using a 1.5 s-running-window median. The black thicker line corresponds to the average over the whole dataset. (TIF) [file pcbi.1012330.s003.tif]

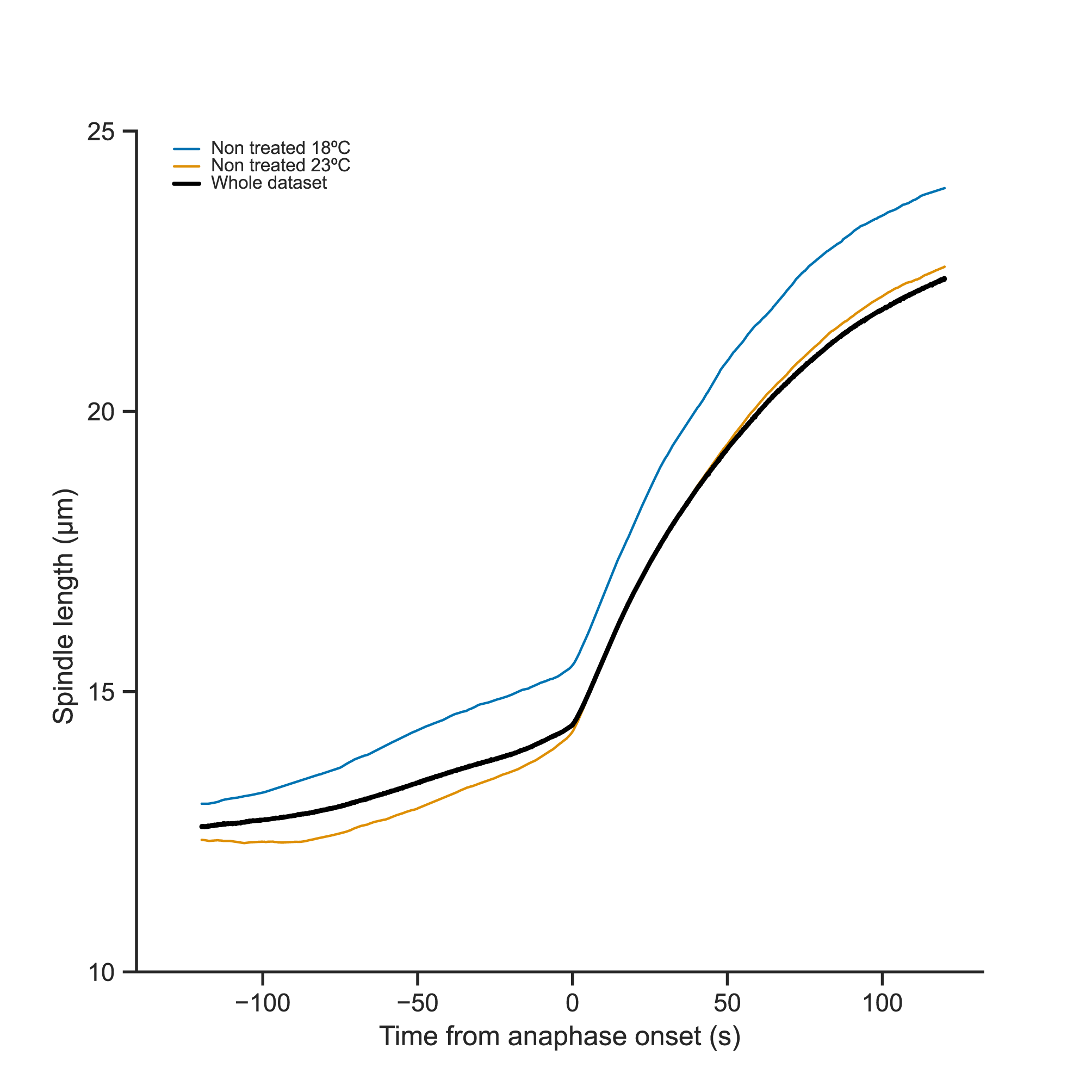

Supplement: S4 Fig — Black thicker line corresponds to the average over the whole dataset, including all conditions. All experiments were done using strain TH27. Tracks were smoothed using a 1.5 s-running-window median. (TIF) [file pcbi.1012330.s004.tif]

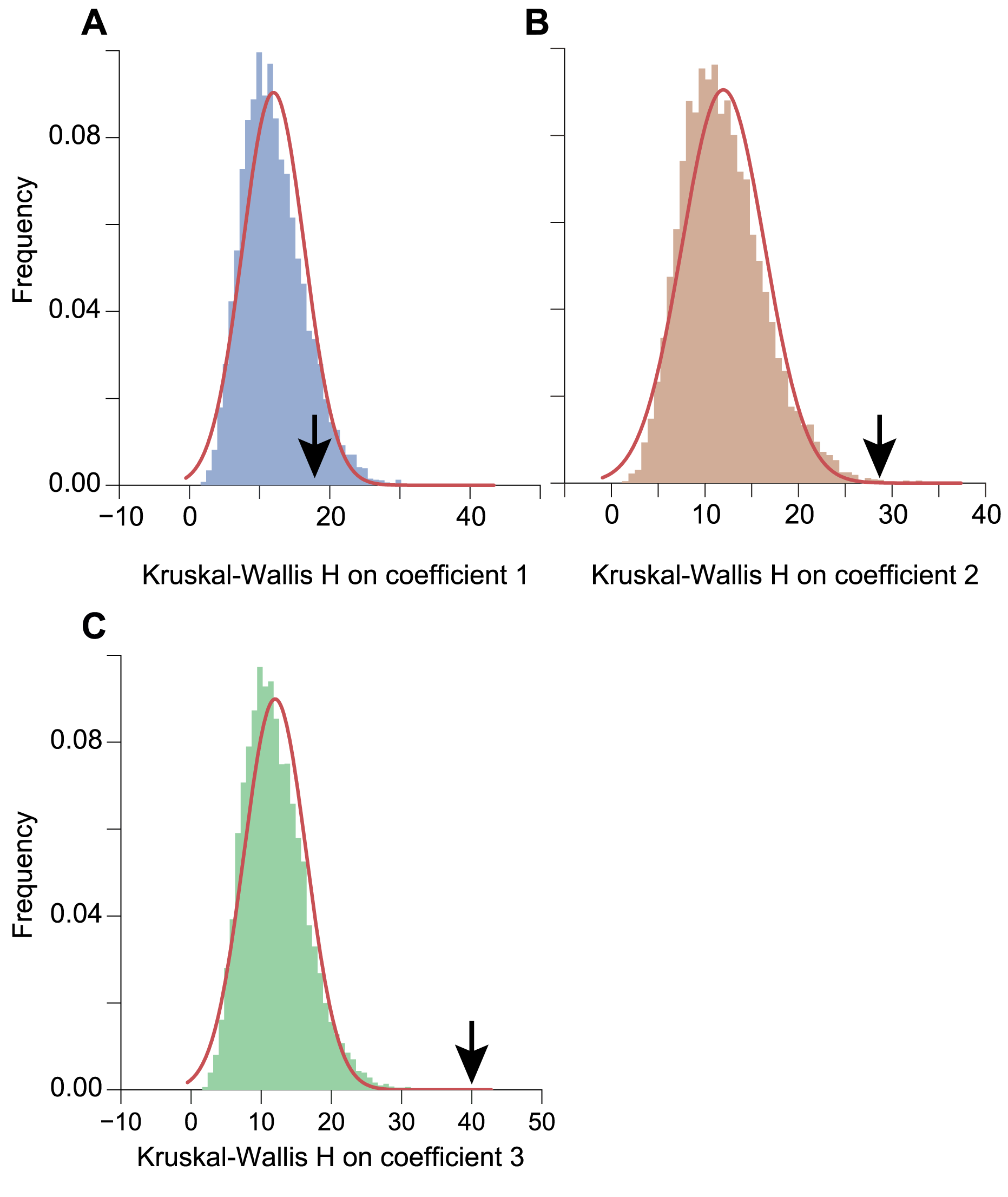

Supplement: S5 Fig — We shuffled the group labels with respect to S1 Table and computed the Kruskal-Wallis H to assess whether conditions from the same group clustered. (A-C) We repeated this computation 10000 times and reported the distribution of H for each coefficient. The arrows indicate the values obtained with true labels for each coefficient, H1 = 17.9, H2 = 28.7 and H3 = 39.9. Red lines depict the maximum-likelihood Gaussian fit. (TIF) [file pcbi.1012330.s005.tif]

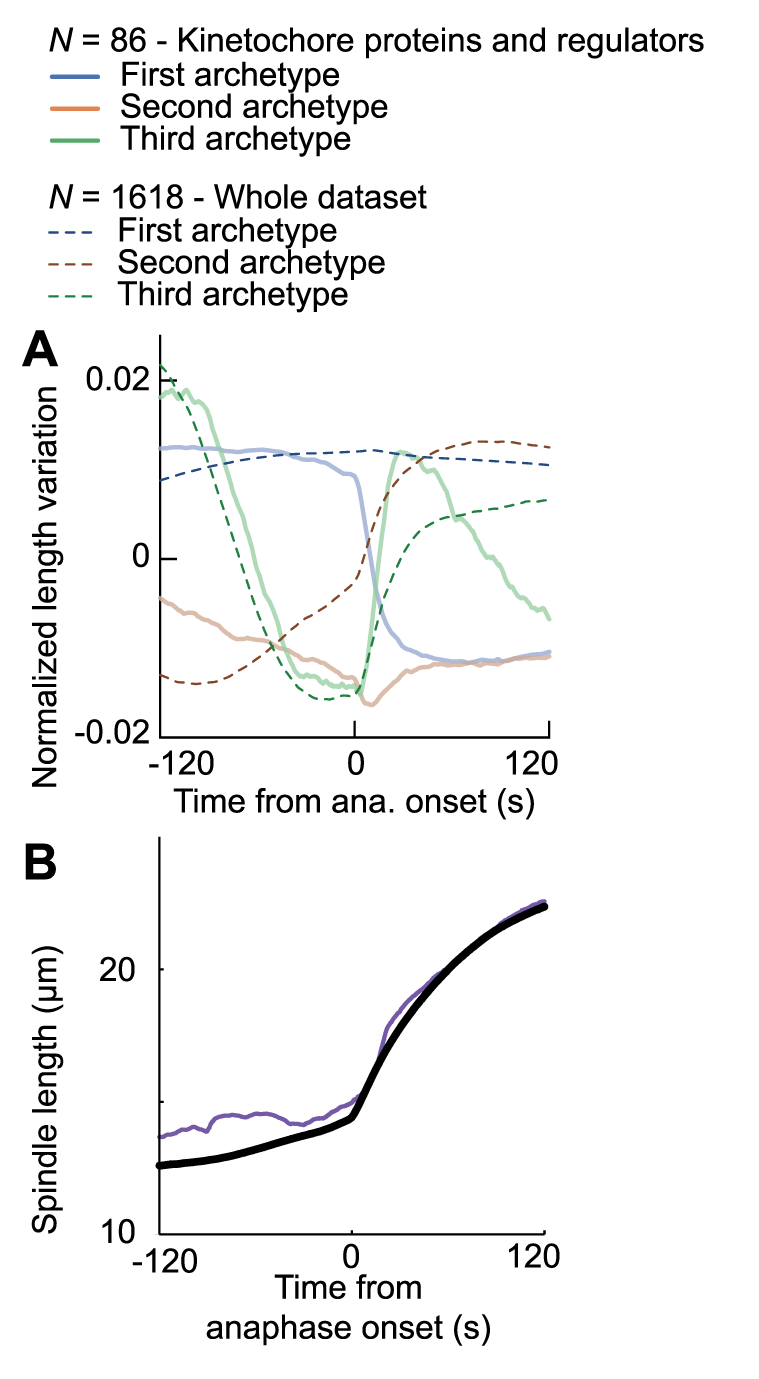

Supplement: S6 Fig — (A) Averages of the three first PCA archetypes computed considering only the embryos from conditions of the group “kinetochore proteins and regulators” (kt) (N=86) and compared to (dashed lines) archetypes extracted from the whole set of conditions (N=1618). The elongation curves were smoothed with a 1.5 s running-median filtering before computing PCA. Explained variance is reported in S5 Table. (B) The corresponding spindle elongation was computed as the median of the average elongation curve among embryos from the same conditions. The track was smoothed using a 1.5 s-running-window median. The black thicker line corresponds to the average over the whole dataset, including all conditions. (TIF) [file pcbi.1012330.s006.tif]

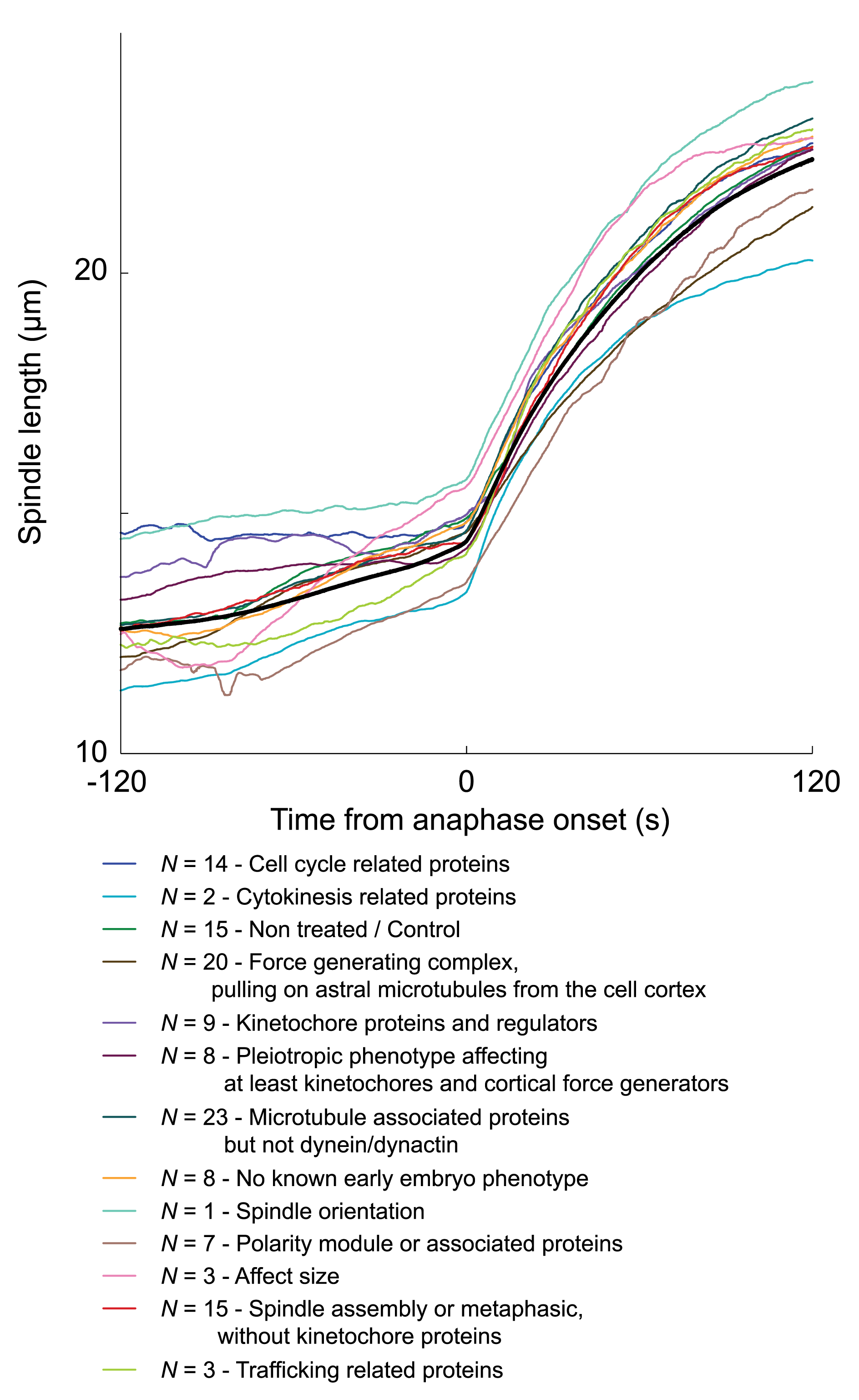

Supplement: S7 Fig — In turn, the elongation for each condition is computed as the average of the curves for each embryo within the condition. Each group track was smoothed using a 1.5 s-running-window median. The black thicker line corresponds to the average over the whole dataset, including all conditions. The corresponding PCA values are reported at Fig 6. (TIF) [file pcbi.1012330.s007.tif]

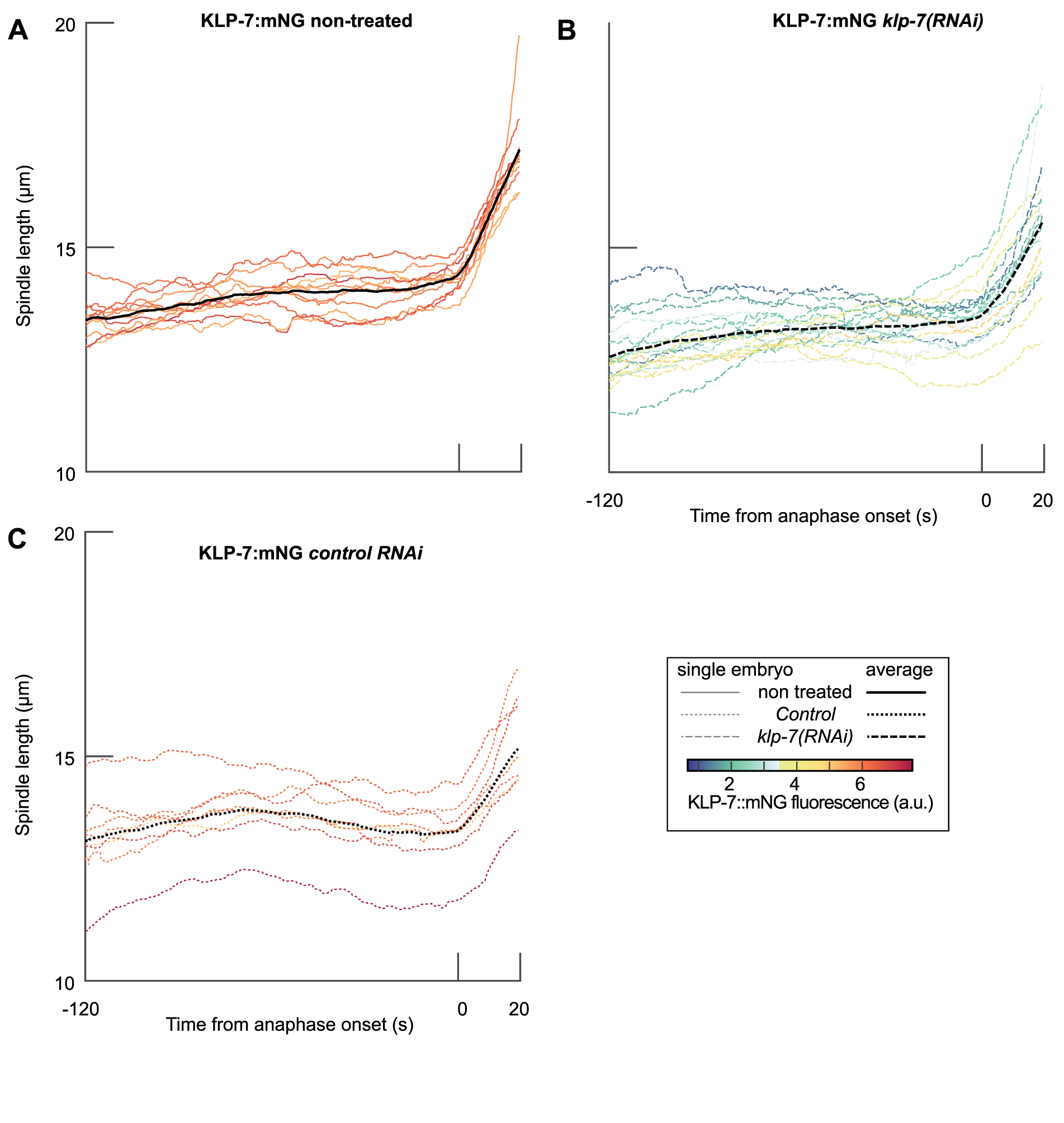

Supplement: S8 Fig — Exemplified through RNAi targetting KLP-7MCAK in KLP-7::mNG background (strain LP447) in three conditions: (A) N=11 non-treated embryos; (B) N=18 klp-7(RNAi)) treated embryos; (C) N=8 control embryos (L4440 treated). The thick lines report the averages of each condition and correspond to the data in Fig 5A. The three conditions reported here were not included in the initial dataset used to generate PCA archetypes. Acquisitions were performed at 18°C. The line colour encodes the fluorescence level of KLP-7::mNG (Methods). (TIF) [file pcbi.1012330.s008.tif]
