# Supplementary material for: Unveiling inter-embryo variability in spindle length over time: Towards quantitative phenotype analysis
Source: PLoS Comput Biol. 2024 Sep 5;20(9):e1012330. doi: 10.1371/journal.pcbi.1012330 (PMC11376571; doi:10.1371/journal.pcbi.1012330)
Supplement: S1 Text — (1) Multilinear fit predicting Farhadifar features by regression on the PCA coefficients and (2) genes causing spindle shortening or limited elongation in late metaphase. (PDF) [file pcbi.1012330.s011.pdf]

Supplemental text for the article "Unveiling Inter-Embryo Variability in Spindle Length over time: towards Quantitative Phenotype Analysis." by Le Cunff et al.

1 Multilinear fit predicting Farhadifar features by regression on the PCA coefficients

We sought a linear relation between the manually selected features reported in [1] and our features without a priori. We first fitted individual embryos elongation curve using the Levenberg-Marquardt algorithm, and the equation $l(t) = l_0 + \frac{l_1}{1+\exp\left(\frac{-(t-t_0)}{\tau}\right)}$ after [1]. The technical variables in this fitting-equation combine into three features: the average spindle length at early metaphase l_0, the final spindle length in anaphase $l_0 + l_1$, and the elongation rate $\alpha = l_1/4\tau$. We excluded the poorly fitted embryos, where the standard error over one of the four fitted variables was higher than 50% of the nominal value (16/1618 embryos) and the 14 additional embryos where fitting produced aberrant values, that was final length larger than 55 µm, the length of the embryo itself, or elongation rate larger than $1\,\mu\mathrm{m\,s}^{-1}$.

Then, we performed a multilinear regression to estimate a linear relation between the average spindle length at early metaphase l_0 and coefficients 1 and 2 in PCA, namely $l_0 = a * C_1 + b * C_2 + c$, with a,b,c the fitting parameters and using ordinary least squares and heteroskedastic consistent variance HC3 in estimating standard errors [2] (Fig 2E) . It led to estimates $a \simeq 19.03 \pm 0.42$ (estimate ±standard error), $b \simeq -22.526 \pm 0.62$ and $c \simeq 12420 \pm 25$, while the R-squared for the model read $R^2 \simeq 0.75$.

We repeated the computation for the final spindle length in anaphase $l_0 + l_1$, and obtained estimates $a \simeq 22.04 \pm 0.65$, $b \simeq 30.71 \pm 1.06$ and $c \simeq 23040 \pm 45$, while $R^2 \simeq 0.58$ (Fig 2F).

We finally investigated the elongation rate $\alpha = l_1/4\tau$, and obtained estimates $a \simeq 0.216 \pm 0.034$, $b \simeq 1.08 \pm 0.11$ and $c \simeq 105.5 \pm 1.6$, while $R^2 \simeq 0.32$ (Fig 2G).

Overall, it suggests that our two first archetypes capture quite well the initial and, to a lower extent, final spindle length as estimated with manually-set features. Meanwhile, the elongation rate likely depends on more archetypes than the two first ones. Consistently, multilinear fits with the three coefficients modestly improve the R^2 for initial and final lengths while being more advantageous for the elongation rate, with values, respectively, $R_3^2 \simeq 0.87$, $R_3^2 \simeq 0.62$ and $R_3^2 \simeq 0.56$.

2 Genes causing spindle shortening or limited elongation in late metaphase

We asked whether genes known to lead to a spindle shortening phenotype during late metaphase display a significantly different PCA projection from their control. We first identified *cls-2*, *tpxl-1* and *bub-1* [3–7]. To extent this list to all related genes in our set, we extracted all genes which share at least 5 common phenotypes with these 3 genes. It indicated *air-2* and *klp-19*. We thus compared the PCA coefficients of individual embryos using the Mann-Whitney test (Suppl Table **??**) and observed a significant difference in the third coefficient, at least in all cases.

Bibliography

[1] Farhadifar R, Baer CF, Valfort AC, Andersen EC, Müller-Reichert T, Delattre M, et al. Scaling, selection, and evolutionary dynamics of the mitotic spindle. Current Biology. 2015;25(6):732–740. doi:10.1016/j.cub.2014.12.060.

[2] MacKinnon JG, White H. Some heteroskedasticity-consistent covariance matrix estimators with improved finite sample properties. Journal of Econometrics. 1985;29(3):305–325. doi:10.1016/0304-4076(85)90158-7.

[3] Ozlu N, Srayko M, Kinoshita K, Habermann B, O'Toole E T, Muller-Reichert T, et al. An essential function of the C. elegans ortholog of TPX2 is to localize activated aurora A kinase to mitotic spindles. Dev Cell. 2005;9(2):237–48. doi:10.1016/j.devcel.2005.07.002.

[4] Lewellyn L, Dumont J, Desai A, Oegema K. Analyzing the effects of delaying aster separation on furrow formation during cytokinesis in the Caenorhabditis elegans embryo. Mol Biol Cell. 2010;21(1):50–62. doi:10.1091/mbc.E09-01-0089.

[5] Cheerambathur DK, Gassmann R, Cook B, Oegema K, Desai A. Crosstalk between microtubule attachment complexes ensures accurate chromosome segregation. Science. 2013;342(6163):1239–42. doi:10.1126/science.1246232.

[6] Edwards F, Maton G, Gareil N, Canman JC, Dumont J. BUB-1 promotes amphitelic chromosome biorientation via multiple activities at the kinetochore. Elife. 2018;7. doi:10.7554/eLife.40690.

[7] Cheeseman IM, MacLeod I, Yates r J R, Oegema K, Desai A. The CENP-F-like proteins HCP-1 and HCP-2 target CLASP to kinetochores to mediate chromosome segregation. Curr Biol. 2005;15(8):771–7. doi:10.1016/j.cub.2005.03.018.
