# Supplementary material for: Unveiling inter-embryo variability in spindle length over time: Towards quantitative phenotype analysis
Source: PLoS Comput Biol. 2024 Sep 5;20(9):e1012330. doi: 10.1371/journal.pcbi.1012330 (PMC11376571; doi:10.1371/journal.pcbi.1012330)
Supplement: S1 Methods — (PDF) [file pcbi.1012330.s012.pdf]

Supplemental methods for the article "Unveiling Inter-Embryo Variability in Spindle Length over time: towards Quantitative Phenotype Analysis." by Le Cunff et al.

1 Evaluating projection methods

In order to unveil the underlying similarities between these various dynamics in spindle elongation, we tested a range of dimension reduction methods, linear, unsupervised and extracting features (Suppl table S4) [1]. For the sake of completeness, we also added two non-linear feature extraction algorithms, namely t-distributed stochastic neighbour embedding (t-SNE) and local linear embedding [2]. For each algorithm and each corresponding projected coordinate, we computed the ratio between inner-condition variability and between-condition variability:

$$\texttt{score} = \frac{\Sigma_{k=1}^{K} n_k(\bar{x_k} - \bar{x})^2}{\Sigma_{k=1}^{K} \Sigma_{x_j \in g_k} (x_j - \bar{x_k})^2}$$

where K is the number of experimental conditions in the whole dataset g_K, $\bar{x_K}$ the average coordinates of the group K, $\bar{x}$ the average over the whole dataset. We also denoted x_j the coordinate of the individual embryo j. We obtained the global score by summing the scores obtained for each dimension. This score is analogous to the statistics used in anova analysis: the higher the score, the more separated the experimental conditions are and the more compact each group of individual experiments within the same experimental condition is in the projection space. It quantifies that experimental replicas tend to cluster together preferentially. Among linear methods that also enable us to interpret the resulting dimensions, Principal Component Analysis (PCA) performed best in clustering our dataset's various experimental conditions (Suppl table S4). Considering PCA, and since our data include replicas of the same experiment, i.e. data points with the same label, we asked whether these are clustered by the projection methods by computing the aforementioned score upon scrambling gene labels; we repeated this assay 10000 times. It produced a Gaussian distributed histogram of mean 0.086 and standard deviation 0.01 (Fig S1D).

2 Mapping features on the projection plan

Each experimental elongation curve is projected using PCA and is described as a point in a 2-dimensional space corresponding to its 2 first PCA coefficients. We concurrently fitted the elongation after [3] and extracted the corresponding features (see main text). To detect a potential gradient of these features in the projection, we fitted a plane in the 3D space (Suppl Text). Then, we extracted the corresponding 2D-gradient of such a plan and depicted it on the PCA plan as an arrow.

3 Machine Learning

To predict the final spindle length (see section 3.3), we used a neural network consisting of seven fully connected layers of 64 neurons each, with a ReLu activation function implemented in Python with the Keras package. This network took a 25 s duration curve (spindle length) as input, i.e. about 800 data points. Alternatively, we put in the coefficients obtained by PCA projection and, in such a case, used a smaller network comprising three hidden layers of 16 neurons each. We computed the spindle length in late anaphase (l_{LA}), as the average of the 300 last data points spanning between 111.7 s and 120 s after anaphase onset and filtered out the embryos giving the 1% shortest and longest values. Then, we performed cross-validation and split the 1584 remaining experiments disregarding experimental conditions: 60% in the training set, 8% in the validation set and 32% in the testing set. We trained using the adam algorithm, mean squared-error loss with batch size equal to 32. We stopped after 200 epochs when using a 25 s duration spindle elongation curve as input and 150 epochs when using PCA projection of elongation over this same interval. Plotting the loss over the validation set did not suggest overfitting. We computed the predicted spindle final length and compared it to the experimental value by computing the Pearson correlation coefficient that we used as the predictive power.

4 Bootstrapping PCA

We used a bootstrap approach to test the robustness of archetypes extracted by PCA. We set to randomly select 500 experiments among the whole dataset of 1618 embryos. It ensures sampling some but few embryos of each of the conditions (Suppl table S1). In doing so, we measured the archetypes' robustness to changes in the dataset size and composition (Fig 3A). Because the high-frequency noise in the elongations is unimportant, we applied a running-window median filtering with size 49 points, corresponding to 1.5 s, to each track before including it in the computation. We made 500 iterations of bootstrapping.

5 Logistic regression on PCA coefficients

We set to use the map of PCA coefficients averaged per condition to predict gene interactions (Suppl File 1), We centred the coefficients on the non-treated condition at the selected temperature. To predict interacting genes, we used a machine learning approach, namely logistic regression. We selected a temperature for our investigation and filtered out the conditions measured at a different temperature. Considering a gene of interest at this temperature, we retrieved its demonstrated and predicted interactors from wormbase [4]; these genes were labelled as interacting. In this instance, we used release WS292, dated from 28 February 2024. The control condition (L4440) and the non-treated conditions were labelled as non-interacting. We then performed a logistic regression on this training set. In all cases reported, we recovered all known/wormbase-predicted interactions for the genes present in our set. Inputing the PCA coefficients of other genes to the trained regression, we can then predict their probability of interacting. When the probability was above 0.9, we considered that these genes were interacting.

Bibliography

[1] Jia W, Sun M, Lian J, Hou S. Feature dimensionality reduction: a review. Complex & Intelligent Systems. 2022;8(3):2663–2693. doi:10.1007/s40747-021-00637-x.

[2] Van der Maaten L, Hinton G. Visualizing data using t-SNE. Journal of machine learning research. 2008;9(11).

[3] Farhadifar R, Ponciano JM, Andersen EC, Needleman DJ, Baer CF. Mutation is a sufficient and robust predictor of genetic variation for mitotic spindle traits in Caenorhabditis elegans. Genetics. 2016;203(4):1859–1870. doi:10.1534/genetics.115.185736.

[4] Harris TW, Antoshechkin I, Bieri T, Blasiar D, Chan J, Chen WJ, et al. WormBase: a comprehensive resource for nematode research. Nucleic Acids Res. 2010;38(Database issue):D463–7. doi:10.1093/nar/gkp952.
