# Supplementary material for: Unveiling inter-embryo variability in spindle length over time: Towards quantitative phenotype analysis
Source: PLoS Comput Biol. 2024 Sep 5;20(9):e1012330. doi: 10.1371/journal.pcbi.1012330 (PMC11376571; doi:10.1371/journal.pcbi.1012330)
Supplement: S1 Table — The fluorescent strains used, carrying possibly mutated genes, are detailed in S2 Table. Target identifies the condition in the figures. RNAi treatments were performed by feeding or injection as detailed in the methods. L4440 corresponds to control conditions for RNAi treatments. Group corresponds to manual tags by function in the one-cell embryo based on papers and known phenotypes in wormbase [79]. Group abbreviations are detailed in Fig 6. (PDF) [file pcbi.1012330.s013.pdf]

Strain	Target	Treatment	Details	Duration	Temperature	Embryos Nb	Group	Reference
TH27	air-1	RNAi-V5J24	feeding-drop	16	18°C	7	spn	this work
JEP31	air-2 cls-2	RNAi-III4J10 mutant or207	feeding-drop	48	15°C	8	kt	this work
JEP31	air-2 gpr-1/2	RNAi-III5C03 mutant or207	feeding-drop	48	15°C	9	fgc	this work
JEP31	air-2 L4440	control RNAi mutant or207	feeding-drop	48	15°C	6	kt	this work
JEP31	air-2 sep-1	RNAi-I7P07 mutant or207	feeding-1mM	24	15°C	11	kt	this work
JEP31	air-2-15C	mutant or207			15°C	8	kt	[1]
JEP31	air-2-25C	mutant or207			25°C	3	kt	[1]
ANA019	ANA019 none	none			18°C	7	ctrl	this work
TH27	ani-2	RNAi II-4P19	feeding-1mM	48	23°C	12	ckn	[2]
TH27	aps-1	RNAi V-4F01	feeding-drop	24	18°C	12	traf	this work
TH27	aspm-1	RNAi I-4F17	feeding-drop	24	18°C	7	map	this work
TH27	bmk-1-18C	RNAi V-9B23	feeding-drop	24	18°C	21	spn	this work
TH27	bmk-1-23C	RNAi V-9B23	feeding-drop	48	23°C	9	spn	this work
TH27	C27D9.1	RNAi II-4A13	feeding-drop	48	23°C	6	size	[2]
TH27	cdk-1	RNAi III-5O13	feeding-drop	12	18°C	4	cc	this work
TH27	cid-1	RNAi III-2P5	feeding-3mM	48	18°C	10	size	this work
TH27	clip-1-18C	RNAi JEP:vec-9	feeding-drop	24	18°C	11	map	this work
JEP32	clip-1-23C	mutant gk470			23°C	9	map	this work
TH27	cls-2-18C	RNAi III-4J10	feeding-drop	24	18°C	12	kt	this work
TH27	cls-2-23C	RNAi III-4J10	feeding-3mM	48	23°C	12	kt	this work
TH27	cls-2-23C	RNAi III-4J10	feeding-drop	24	23°C	9	kt	this work
TH27	csnk-1	RNAi I-5K03	feeding-1mM	24	23°C	6	pol	this work
TH27	daf-7	RNAi III-1C04	feeding-drop	24	18°C	6	nop	this work
TH27	dhc-1	RNAi I-1P04	feeding-2mM	20	18°C	5	kt+fgc	this work
TH27	dli-1	RNAi IV-7A05	feeding-drop	12	18°C	8	kt+fgc	this work
TH27	dli-1	RNAi IV-7A05	feeding-drop	24	18°C	5	kt+fgc	[3]
TH27	dnc-1	RNAi IV-5D24	feeding-drop	24	18°C	16	kt+fgc	this work
TH27	dyci-1	RNAi IV-3G8	feeding-2mM	16	18°C	6	kt+fgc	[3]
JEP27	dyci-1 ebp-2	RNAi IV-3G8 mutant gk756	feeding-2mM	16	18°C	8	kt+fgc	this work
JEP15	dylt-1-18C	RNAi I-3J11	feeding-3mM	24	18°C	11	kt+fgc	this work
JEP15	dylt-1-18C	RNAi I-3J11	feeding-3mM	48	18°C	12	kt+fgc	this work
JEP15	dylt-1-18C	RNAi I-3J11	feeding-drop	24	18°C	10	kt+fgc	this work
TH27	dylt-1-20C	RNAi I-3J11	feeding-drop	24	20°C	5	kt+fgc	this work
TH27	dyrb-1	RNAi II-6F21	feeding-drop	24	18°C	12	kt+fgc	this work
TH27	ebp-1	RNAi cenix:236-e2	feeding-2mM	24	18°C	7	map	this work
TH27	ebp-1	RNAi cenix:236-e2	feeding-3mM	48	18°C	11	map	[3]
TH27	ebp-1	RNAi JEP:vec-33	feeding-2mM	24	18°C	9	map	this work
TH27	ebp-1	RNAi JEP:vec-33	feeding-3mM	48	18°C	10	map	this work
JEP27	ebp-1 ebp-2	RNAi cenix:236-e2 mutant gk756	feeding-2mM	24	18°C	9	map	this work
JEP27	ebp-1 ebp-2	RNAi cenix:236-e2 mutant gk756	feeding-3mM	48	18°C	12	map	[3]

Strain	Target	Treatment	Details	Duration	Temperature	Embryos Nb	Group	Reference
JEP27	ebp-1 ebp-2	RNAi JEP:vec-33 mutant gk756	feeding-2mM	24	18°C	4	map	this work
JEP27	ebp-1 ebp-2	RNAi JEP:vec-33 mutant gk756	feeding-3mM	48	18°C	9	map	this work
JEP27	ebp-1 ebp-2 ebp-3	RNAi JEP:vec-35 mutant gk756	feeding-2mM	24	18°C	9	map	this work
JEP27	ebp-1 ebp-2 ebp-3	RNAi JEP:vec-35 mutant gk756	feeding-3mM	48	18°C	9	map	[3]
TH27	ebp-1 ebp-3	RNAi JEP:vec-35	feeding-2mM	24	18°C	8	map	this work
TH27	ebp-1 ebp-3	RNAi JEP:vec-35	feeding-3mM	48	18°C	5	map	[3]
TH27	ebp-2-18C	RNAi II-7F10	feeding-3mM	48	18°C	8	map	[3]
TH27	ebp-2-18C	RNAi II-7F10	feeding-drop	24	18°C	6	map	this work
TH27	ebp-2-20C	RNAi II-7F10	feeding-drop	18	20°C	4	map	this work
JEP46	ebp2clip1	mutant gk756 mutant gk470			18°C	8	map	this work
TH27	efa-6-18C	RNAi IV-6P21	feeding-drop	24	18°C	13	fgc	this work
TH27	efa-6-23C	RNAi IV-6P21	feeding-drop	24	23°C	4	fgc	this work
TH27	egl-5	RNAi III-4I05	feeding-drop	24	18°C	7	nop	this work
TH27	F21H12.2	RNAi II-4F17	feeding-3mM	48	23°C	7	nop	this work
TH27	goa-1	RNAi I-3P15	feeding-drop	24	18°C	15	fgc	this work
TH65	goa-1 gpa-16	RNAi III-1K08	feeding-3mM	48	23°C	9	fgc	this work
TH27	gpa-16	RNAi II-9I-18	feeding-drop	24	18°C	8	fgc	this work
TH27	gpb-1	RNAi II-8A5	feeding-1mM	24	18°C	8	fgc	this work
TH27	gpr-1 gpr-2-18C	RNAi III-4J09 RNAi III-4J09	feeding-drop	24	18°C	8	fgc	[4]
TH27	gpr-1 gpr-2-23C	RNAi III-4J09 RNAi III-4J09	feeding-1mM	4-6	23°C	15	fgc	[2]
TH65	gpr-1 gpr-2-23C	RNAi III-4J09 RNAi III-4J09	feeding-4mM	48	23°C	7	fgc	[2]
TH27	ima-3	RNAi IV-3H04	feeding-1mM	34	23°C	5	size	[2]
JEP1	JEP1 klp-13-18C	mutant tm3737			18°C	19	map	this work
JEP13	JEP13gpr-1	mutant ok2126			18°C	7	fgc	[4]
JEP14	JEP14gpr-2-18C	mutant ok1179			18°C	6	fgc	[4]
JEP14	JEP14gpr-2-23C	mutant ok1179			23°C	6	fgc	this work
JEP15	JEP15 L4440	control RNAi	feeding-3mM	24	18°C	11	ctrl	this work
JEP15	JEP15 L4440	control RNAi	feeding-drop	24	18°C	7	ctrl	this work
JEP15	JEP15 none	none			23°C	6	ctrl	[2]
JEP27	JEP27 none ebp-2	none			18°C	8	map	this work
JEP29	JEP29 none 15C	none			15°C	9	ctrl	this work
JEP29	JEP29 none 25C	none			25°C	6	ctrl	this work
JEP3	JEP3 gpr-1	mutant ok2126			18°C	8	fgc	this work
JEP4	JEP4 gpr-2-18C	mutant tm964			18°C	9	fgc	this work
JEP4	JEP4 gpr-2-23C	mutant tm964			23°C	6	fgc	this work
JEP5	JEP5 mbk-2	mutant ne992			18°C	23	cc	this work
JEP6	JEP6 lin-5-18C	mutant ev571			18°C	6	fgc	this work
TH27	klp-10	RNAi IV-3N16	feeding-drop	24	18°C	2	map	this work
TH27	klp-11	RNAi IV-4O14	feeding-drop	24	18°C	8	map	this work

Strain	Target	Treatment	Details	Duration	Temperature	Embryos Nb	Group	Reference
TH27	klp-12	RNAi IV-6G03	feeding-drop	24	18°C	6	map	this work
TH27	klp-13-18C	RNAi X-3C14	feeding-3mM	24	18°C	7	map	this work
TH27	klp-13-18C	RNAi X-3C14	feeding-drop	24	18°C	6	map	this work
TH27	klp-13-23C	RNAi X-3C14	feeding-3mM	24	23°C	11	map	this work
TH27	klp-15	RNAi I-2F15	feeding-drop	24	18°C	9	spn	this work
TH27	klp-16	RNAi I-4P13	feeding-3mM	48	18°C	9	spn	this work
TH27	klp-16	RNAi I-4P13	feeding-drop	24	18°C	15	spn	this work
TH27	klp-17	RNAi II-7F24	feeding-drop	24	18°C	3	spn	this work
TH27	klp-17	RNAi II-7H02	feeding-drop	24	18°C	3	spn	this work
TH27	klp-18	RNAi IV-3O14	feeding-drop	24	18°C	8	spn	this work
TH27	klp-19	RNAi III-623	feeding-drop	24	18°C	10	kt	this work
TH27	klp-20	RNAi JEP:vec-06	feeding-drop	24	18°C	9	map	this work
TH27	klp-3	RNAi II-5H18	feeding-drop	24	18°C	7	kt	this work
TH27	klp-4	RNAi X-2K05	feeding-drop	24	18°C	8	map	this work
TH27	klp-6	RNAi III-2J07	feeding-drop	24	18°C	3	map	this work
TH27	klp-7	RNAi III-5B24	feeding-drop	24	18°C	9	spn	this work
TH27	klp-7	RNAi III-5B24	feeding-drop	36	18°C	6	spn	this work
TH27	klp-7	RNAi III-5B24	feeding-drop	48	18°C	4	spn	this work
TH27	klp-8	RNAi X-2I-17	feeding-drop	24	18°C	8	nop	this work
JEP27	L4440 ebp-2	control RNAi mutant gk756	feeding-2mM	24	18°C	10	map	this work
JEP27	L4440 ebp-2	control RNAi mutant gk756	feeding-3mM	48	18°C	8	map	this work
TH27	L4440-18C	control RNAi	feeding-drop	24	18°C	13	ctrl	this work
TH27	L4440-18C	control RNAi	feeding-drop	48	18°C	6	ctrl	[3]
TH27	L4440-18C	control RNAi	feeding-1mM	6-10	18°C	10	ctrl	this work
TH27	L4440-18C	control RNAi	feeding-1mM	6-10	18°C	3	ctrl	this work
TH27	L4440-18C	control RNAi	feeding-3mM	24	18°C	5	ctrl	this work
TH27	L4440-18C	control RNAi	feeding-3mM	48	18°C	8	ctrl	this work
TH27	L4440-18C	control RNAi	feeding-drop	24	18°C	9	ctrl	this work
TH27	L4440-18C	control RNAi	feeding-drop	24	18°C	9	ctrl	this work
TH27	L4440-20C	control RNAi	feeding-drop	24	20°C	3	ctrl	this work
TH27	L4440-23C	control RNAi	feeding-1mM	24	23°C	11	ctrl	[2]
TH27	L4440-23C	control RNAi	feeding-1mM	48	23°C	4	ctrl	this work
TH27	L4440-23C	control RNAi	feeding-1mM	6	23°C	14	ctrl	[2]
TH27	L4440-23C	control RNAi	feeding-3mM	48	23°C	28	ctrl	this work
TH27	L4440-23C	control RNAi	feeding-drop	24	23°C	8	ctrl	this work
TH27	L4440-23C	control RNAi	feeding-1mM	6-10	23°C	8	ctrl	[2]
TH27	L4440-23C	control RNAi	feeding-4mM	24	23°C	6	ctrl	this work
TH27	L4440-23C	control RNAi	feeding-drop	24	23°C	9	ctrl	this work
TH27	L4440-23C	control RNAi	feeding-drop	6	23°C	9	ctrl	this work
TH65	L4440-23C	control RNAi	feeding-4mM	48	23°C	10	ctrl	[2]
TH27	let-99	RNAi JEP:vec-37	feeding-3mM	24	23°C	8	pol	[2]
TH27	let-99	RNAi JEP:vec-37	feeding-drop	24	23°C	8	pol	this work
TH27	lin-5-18C	RNAi II-5J10	feeding-drop	24	18°C	12	fgc	this work
TH27	lin-5-18C	RNAi II-5J10	injection	40	18°C	10	fgc	[4]
TH27	lin-5-23C	RNAi II-5J10	feeding-3mM	16	23°C	11	fgc	this work

Strain	Target	Treatment	Details	Duration	Temperature	Embryos Nb	Group	Reference
TH27	lov-1	RNAi II-7K23	feeding-drop	24	18°C	8	ctrl	this work
TH27	lov-1	RNAi II-7K23	injection	41	18°C	10	ctrl	[4]
TH27	lrg-1	RNAi III-5G03	feeding-drop	24	18°C	5	nop	this work
TH27	mab-5	RNAi III-4I09	feeding-drop	24	18°C	5	nop	this work
TH27	mbk-2	RNAi cenix:10-e8	feeding-drop	24	18°C	7	cc	this work
TH27	nmy-2	RNAi I-3L24	feeding-drop	24	18°C	7	pol	[4]
TH27	none-18C	none			18°C	58	ctrl	[4]
TH27	none-23C	none			23°C	71	ctrl	[2]
TH27	osm-3	RNAi III-2J07	feeding-drop	24	18°C	6	map	this work
TH65	par-2	RNAi III-1K08	feeding-3mM	48	23°C	5	pol	[5]
TH65	par-2	RNAi III-1K08	feeding-3mM	24	23°C	9	pol	this work
TH27	par-3	RNAi III-3A01	feeding-1mM	24	23°C	15	pol	[2]
TH27	par-3	RNAi III-3A01	feeding-1mM	30	23°C	6	pol	this work
TH27	par-4	RNAi bact-16	feeding-drop	24	18°C	15	pol	this work
TH27	pkd-2	RNAi IV-7P23	feeding-drop	24	18°C	6	nop	this work
TH27	plk-1-18C	RNAi III-4C10	feeding-drop	16	18°C	1	cc	this work
TH27	plk-1-18C	RNAi III-4E08	feeding-drop	16	18°C	2	cc	this work
TH27	plk-1-23C	RNAi III-4C10	feeding-drop	6	23°C	10	cc	this work
TH27	ptl-1	RNAi III-1A12	feeding-drop	24	18°C	12	map	this work
TH27	ptl-1	RNAi III-1A24	feeding-drop	24	18°C	13	map	this work
TH27	ptl-1	RNAi III-1A24	feeding-drop	36	18°C	10	map	this work
TH27	spat-1	RNAi II-9O07	feeding-drop	24	18°C	6	cc	this work
TH27	spd-1	RNAi I-7D17	feeding-drop	24	18°C	7	spn	this work
TH27	spd-2-18C	RNAi I-4O08	feeding-1mM	6-10	18°C	19	spn	this work
TH27	spd-2-23C	RNAi I-4O08	feeding-1mM	6	23°C	9	spn	[2]
TH27	spn-4	RNAi JEP:vec-11	feeding-drop	24	18°C	8	ori	this work
JEP16	such-1	mutant h1960			23°C	11	cc	[2]
JEP16	such-1 dylt-1	mutant h1960 RNAi I-3J11	feeding-3mM	24	18°C	9	cc	this work
JEP16	such-1 dylt-1	mutant h1960 RNAi I-3J11	feeding-3mM	48	18°C	10	cc	this work
JEP16	such-1 dylt-1	mutant h1960 RNAi I-3J11	feeding-3mM	96	18°C	2	cc	this work
JEP16	such-1 L4440	mutant h1960 - control RNAi	feeding-3mM	24	18°C	9	cc	this work
JEP16	such-1 L4440	mutant h1960 - control RNAi	feeding-drop	24	18°C	8	cc	this work
JEP10	such-1 mdf-1	mutants h1960 gk2			18°C	22	cc	this work
JEP17	such-1 mdf-1 dylt-1	mutants h1960 gk2 RNAi I-3J11	feeding-3mM	48	18°C	7	cc	this work
JEP17	such-1 mdf-1 L4440	mutants h1960 gk2 - control RNAi	feeding-3mM	24	18°C	8	cc	this work
TH27	tac-1	RNAi II-9C20	feeding-drop	24	18°C	7	map	this work
TH102	TH102 none	none			18°C	3	ctrl	this work
TH231	TH231 none	none			18°C	21	ctrl	this work
TH65	TH65 lin-5-23C	RNAi II-5J10	feeding-3mM	24	23°C	9	fgc	[5]
TH65	TH65 none	none			23°C	7	ctrl	[2]

Strain	Target	Treatment	Details	Duration	Temperature	Embryos Nb	Group	Reference
TH65	TH65 par-3	RNAi III-3A01	feeding-3mM	48	23°C	11	pol	[2]
TH27	tpxl-1-18C	RNAi I-7J23	feeding-drop	24	18°C	4	spn	this work
TH27	tpxl-1-18C	RNAi I-7L01	feeding-drop	24	18°C	3	spn	this work
TH27	tpxl-1-23C	RNAi I-7L01	feeding-drop	24	23°C	12	spn	this work
TH27	ubxn-2	RNAi JEP:vec-7	feeding-drop	24	18°C	3	cc	this work
TH27	unc-104	RNAi II-5G20	feeding-drop	24	18°C	7	traf	this work
TH27	unc-116	RNAi III-4O16	feeding-drop	24	18°C	5	traf	this work
TH27	unc-59	RNAi I-6N04	feeding-drop	24	18°C	13	ckn	this work
TH27	vab-8	RNAi V-8O01	feeding-drop	24	18°C	2	nop	this work
TH27	vab-8	RNAi V-8O05	feeding-drop	24	18°C	3	nop	this work
TH27	zen-4	RNAi IV-3I07	feeding-drop	24	18°C	7	spn	this work
TH27	zyg-11	RNAi II-5N06	feeding-drop	24	18°C	6	cc	this work
TH27	zyg-9	RNAi II-6M11	feeding-3mM	4	18°C	11	spn	this work
TH27	zyg-9	RNAi II-6M11	feeding-drop	24	18°C	3	spn	[4]

Bibliography

[1] Severson AF, Hamill DR, Carter JC, Schumacher J, Bowerman B. The aurora-related kinase AIR-2 recruits ZEN-4/CeMKLP1 to the mitotic spindle at metaphase and is required for cytokinesis. Curr Biol. 2000;10(19):1162–71. doi:10.1016/s0960-9822(00)00715-6.

[2] Bouvrais H, Chesneau L, Pastezeur S, Fairbrass D, Delattre M, Pecreaux J. Microtubule Feedback and LET-99-Dependent Control of Pulling Forces Ensure Robust Spindle Position. Biophys J. 2018;115(11):2189–2205. doi:10.1016/j.bpj.2018.10.010.

[3] Rodriguez-Garcia R, Chesneau L, Pastezeur S, Roul J, Tramier M, Pécréaux J. The polarity-induced force imbalance in Caenorhabditis elegans embryos is caused by asymmetric binding rates of dynein to the cortex. Am Soc Cell Biol. 2018;29(26):3093–3104. doi:10.1091/mbc.E17-11-0653.

[4] Pécréaux J, Redemann S, Alayan Z, Mercat B, Pastezeur S, Garzon-Coral C, et al. The Mitotic Spindle in the One-Cell C. elegans Embryo Is Positioned with High Precision and Stability. Biophysical Journal. 2016;111(8):1773–1784. doi:10.1016/j.bpj.2016.09.007.

[5] Bouvrais H, Chesneau L, Le Cunff Y, Fairbrass D, Soler N, Pastezeur S, et al. The coordination of spindle-positioning forces during the asymmetric division of the Caenorhabditis elegans zygote. EMBO Rep. 2021;22(5):e50770. doi:10.15252/embr.202050770.
