# Supplementary material for: Unveiling inter-embryo variability in spindle length over time: Towards quantitative phenotype analysis
Source: PLoS Comput Biol. 2024 Sep 5;20(9):e1012330. doi: 10.1371/journal.pcbi.1012330 (PMC11376571; doi:10.1371/journal.pcbi.1012330)
Supplement: S2 Table — Original strains are referenced by each of the crossed strains, whereas previously disclosed ones are referenced by the corresponding publication. (PDF) [file pcbi.1012330.s014.pdf]

Strain	Genotype	Crossing	Origin and Reference
ANA019	C. *briggsae pie-1::Ce-tbg-1::GFP; Ce-sid-2*		[1]
JEP1	*unc-119(ed3) III; ddIs6 [Ppie-1::GFP::tbg-1; unc-119(+)] V; klp-13(tm3737) X*	TH27 x TM3737	[2, 3]
JEP3	*ddIs6 [Ppie-1::GFP::tbg-1; unc-119(+)] V; gpr-1(ok2126) III*	TH27 x VC1670	[2, 4]
JEP4	*ddIs6 [Ppie-1::GFP::tbg-1; unc-119(+)] V; gpr-2(tm964) III*	TH27 x TM964	[2, 3]
JEP5	*unc-119(ed3) III; ddIs6 [Ppie-1::GFP::tbg-1; unc-119(+)] V; mbk-2(ne992) IV*	TH27 x WM73	[2, 5]
JEP6	*unc-119(ed3) III; ddIs6 [Ppie-1::GFP::tbg-1; unc-119(+)] V; lin-5(ev571) II*	TH27 x SV124	[2, 6]
JEP10	*such-1(h1960) III; unc-46(e177) mdf-1(gk2) V. ddIs180[WRM062cF05 spd-2::2xTY1 GFP FRT 3xFlag;unc-119(+)]*	KR4012 x TH231	[7, 8]
JEP13	*gpr-1(ok2126) III. unc-119(ed3) III (?); ddIs6 [Ppie-1::GFP::tbg-1; unc-119(+)] V*	TH27 x TH290	[9]
JEP14	*gpr-2(ok1179) III. unc-119(ed3) III (?); ddIs6 [Ppie-1::GFP::tbg-1; unc-119(+)] V*	TH27 x TH291	[9]
JEP15	*ddIs180[WRM062cF05 spd-2:: 2xTY1 GFP FRT 3xFlag;unc-119(+)] ltIs37 [pie-1p::mCherry::his-58 (pAA64) + unc-119(+)] IV*	JEP10 x OD56	[10]
JEP16	*such-1(h1960) III; ddIs180[WRM062cF05 spd-2:: 2xTY1 GFP FRT3xFlag;unc-119(+)]; ltIs37 [pie-1p::mCherry::his-58 (pAA64) + unc-119(+)] IV*	JEP10 x OD56	[10]
JEP17	*such-1(h1960) III; unc-46(e177) mdf-1(gk2) V. ddIs180[WRM062cF05 spd-2:: 2xTY1 GFP FRT 3xFlag;unc-119(+)] ltIs37 [pie-1p::mCherry::his-58 (pAA64)/unc-119(+)] IV*	JEP10 x OD56	[10]
JEP25	*air-2(or207) unc-13(e51) I ddIs153[WRM064C_D03::unc-119-Nat([18578] knl-1::2xTY1wEGFP3xflag)]*	EU707 x TH243	[11, 12]
JEP27	*ebp-2(gk756) II. ddIs6 [Ppie-1::GFP::tbg-1; unc-119(+)] V*	TH27 x VC1614	[13]
JEP29	*unc-119(ed3)III; ddIs153[WRM064C_D03::unc-119-Nat([18578] knl-1::2xTY1wEGFP3xflag); ddIs180[WRM062cF05 spd-2:: 2xTY1 GFP FRT 3xFlag;unc-119(+)]*	TH231 x TH243	[8, 12]
JEP31	*air-2(or207) unc-13(e51) I; ddIs153[WRM064C_D03::unc-119-Nat([18578] knl-1::2xTY1wEGFP3xflag); ddIs180[WRM062cF05 spd-2:: 2xTY1 GFP FRT 3xFlag;unc-119(+)]*	JEP25 x JEP29	[11]

Strain	Genotype	Crossing	Origin and Reference
JEP32	*clip-1(gk470) III; ddIs6 [Ppie-1::GFP::tbg-1; unc-119(+)] V*	TH27 x VC1071	[13]
JEP46	*ebp-2(gk756) II; clip-1(gk470) III; ddIs6 [Ppie-1::GFP::tbg-1; unc-119(+)] V*	JEP27 x JEP32	this work
TH27	*unc-119(ed3) III; ddIs6 [Ppie-1::GFP::tbg-1; unc-119(+)] V*		[2]
TH65	*unc-119(ed3); ddIs15 [pPIE-1::YFP::tba-2(genomic);unc-119(+)]*		[14]
TH102	*N-YFP::spd-5*		[15]
TH231	*unc-119(ed3)III; ddIs180[WRM062cF05 spd-2:: 2xTY1 GFP FRT 3xFlag;unc-119(+)]*		[8]
LP447	*klp-7(cp178[klp-7::mNG-C1^3xFlag]) III*		[16]

Bibliography

[1] Riche S. Etude comparative du positionnement du fuseau mitotique dans les espèces de C.elegans et C. briggsae [Thesis]. Ecole normale supérieure de Lyon; 2015.

[2] Oegema K, Desai A, Rybina S, Kirkham M, Hyman AA. Functional analysis of kinetochore assembly in Caenorhabditis elegans. The Journal of Cell Biology. 2001;153(6):1209–26. doi:10.1083/jcb.153.6.1209.

[3] elegans Deletion Mutant Consortium C. Large-scale screening for targeted knockouts in the Caenorhabditis elegans genome. G3 (Bethesda). 2012;2(11):1415–25. doi:10.1534/g3.112.003830.

[4] Barstead R, Moulder G, Cobb B, Frazee S, Henthorn D, Holmes J, et al. Large-Scale Screening for Targeted Knockouts in the Caenorhabditis elegans Genome. G3-Genes Genomes Genetics. 2012;2(11):1415–1425. doi:10.1534/g3.112.003830.

[5] Pang KM, Ishidate T, Nakamura K, Shirayama M, Trzepacz C, Schubert CM, et al. The minibrain kinase homolog, mbk-2, is required for spindle positioning and asymmetric cell division in early C. elegans embryos. Dev Biol. 2004;265(1):127–39. doi:10.1016/j.ydbio.2003.09.024.

[6] Lorson MA, Horvitz HR, van den Heuvel S. LIN-5 is a novel component of the spindle apparatus required for chromosome segregation and cleavage plane specification in Caenorhabditis elegans. Journal of Cell Biology. 2000;148(1):73–86. doi:DOI 10.1083/jcb.148.1.73.

[7] Tarailo M, Kitagawa R, Rose AM. Suppressors of spindle checkpoint defect (such) mutants identify new mdf-1/MAD1 interactors in Caenorhabditis elegans. Genetics. 2007;175(4):1665–79. doi:10.1534/genetics.106.067918.

[8] Decker M, Jaensch S, Pozniakovsky A, Zinke A, O'Connell KF, Zachariae W, et al. Limiting amounts of centrosome material set centrosome size in C. elegans embryos. Curr Biol. 2011;21(15):1259–67. doi:S0960-9822(1100647-6 [pii] 10.1016/j.cub.2011.06.002.

[9] Pécréaux J, Redemann S, Alayan Z, Mercat B, Pastezeur S, Garzon-Coral C, et al. The Mitotic Spindle in the One-Cell C. elegans Embryo Is Positioned with High Precision and Stability. Biophysical Journal. 2016;111(8):1773–1784. doi:10.1016/j.bpj.2016.09.007.

[10] Bouvrais H, Chesneau L, Pastezeur S, Fairbrass D, Delattre M, Pecreaux J. Microtubule Feedback and LET-99-Dependent Control of Pulling Forces Ensure Robust Spindle Position. Biophys J. 2018;115(11):2189–2205. doi:10.1016/j.bpj.2018.10.010.

[11] Severson AF, Hamill DR, Carter JC, Schumacher J, Bowerman B. The aurora-related kinase AIR-2 recruits ZEN-4/CeMKLP1 to the mitotic spindle at metaphase and is required for cytokinesis. Curr Biol. 2000;10(19):1162–71. doi:10.1016/s0960-9822(00)00715-6.

[12] Sarov M, Murray JI, Schanze K, Pozniakovski A, Niu W, Angermann K, et al. A genome-scale resource for in vivo tag-based protein function exploration in C. elegans. Cell. 2012;150(4):855–66. doi:10.1016/j.cell.2012.08.001.

[13] Rodriguez-Garcia R, Chesneau L, Pastezeur S, Roul J, Tramier M, Pécréaux J. The polarity-induced force imbalance in Caenorhabditis elegans embryos is caused by asymmetric binding rates of dynein to the cortex. Am Soc Cell Biol. 2018;29(26):3093–3104. doi:10.1091/mbc.E17-11-0653.

[14] Srayko M, Kaya A, Stamford J, Hyman AA. Identification and characterization of factors required for microtubule growth and nucleation in the early C. elegans embryo. Developmental Cell. 2005;9(2):223–236. doi:10.1016/j.devcel.2005.07.003.

[15] Greenan G, Brangwynne CP, Jaensch S, Gharakhani J, Jülicher F, Hyman AA. Centrosome Size Sets Mitotic Spindle Length in Caenorhabditis elegans Embryos. Current Biology. 2010;20(4):353–358. doi:10.1016/j.cub.2009.12.050.

[16] Heppert JK, Pani AM, Roberts AM, Dickinson DJ, Goldstein B. A CRISPR Tagging-Based Screen Reveals Localized Players in Wnt-Directed Asymmetric Cell Division. Genetics. 2018;208(3):1147–1164. doi:10.1534/genetics.117.300487.
