# Supplementary material for: Unveiling inter-embryo variability in spindle length over time: Towards quantitative phenotype analysis
Source: PLoS Comput Biol. 2024 Sep 5;20(9):e1012330. doi: 10.1371/journal.pcbi.1012330 (PMC11376571; doi:10.1371/journal.pcbi.1012330)
Supplement: S3 Table — (PDF) [file pcbi.1012330.s015.pdf]

Name	Target	Primers (forward / reverse)	Reference
JEP:vec-6	*klp-20*	5'-AGTACATTCCGGTGGAGCAC-3'<br>5'-TAGGCAATTGCTTTGAGCTG-3'	this work
JEP:vec-7	*ubxn-2*	5'-AAAGTGAACCGCCACCAC-3'<br>5'-CAACATTTCCCAAACGGACT-3'	this work
JEP:vec-9	*clip-1*	5'-TCCCGATGGTTCAATCAGTTT-3'<br>5'-GCATCCTCCCTTTCTTTTCA-3'	this work
JEP:vec-11	*spn-4*	5'-GAGCGACACCAACCCGCAGA-3'<br>5'-ATCTGGTCACGAAGATGATGTGGGA-3'	this work
JEP:vec-37	*let-99*	5'-CCACCAAAGGCAAG-3'<br>5'-AAGTGATCTGTTCAAAATCTTCGGA-3'	this work
