# Supplementary material for: Unveiling inter-embryo variability in spindle length over time: Towards quantitative phenotype analysis
Source: PLoS Comput Biol. 2024 Sep 5;20(9):e1012330. doi: 10.1371/journal.pcbi.1012330 (PMC11376571; doi:10.1371/journal.pcbi.1012330)
Supplement: S4 Table — We compared various projection methods by assessing their ability to cluster replicas while separating experiments corresponding to distinct treatments, using the score described in S1 Methods. We also included some non-linear/local methods for the sake of completeness, although they will not enable the interpretability expected in our specifications. Higher scores mean that the projection method performs better. The last row correspond to the average upon 10000 repeats of shuffling experiment-labels and computing the score. (PDF) [file pcbi.1012330.s016.pdf]

**Dimension reduction method**	**Score**
Principal Component Analysis (PCA)	1.86
Non-metric Multidimensional scaling	1.71
Multidimensional Scaling	1.68
t-distributed stochastic neighbour embedding (t-SNE, non-linear)	1.36
Local Linear Embedding (non-linear)	2.26
Independent Component Analysis	1.43
Factor Analysis	1.41
Truncated Singular-Value Decomposition	1.74
Principal Component Analysis with scrambled labels	0.086
