# Supplementary material for: Unveiling inter-embryo variability in spindle length over time: Towards quantitative phenotype analysis
Source: PLoS Comput Biol. 2024 Sep 5;20(9):e1012330. doi: 10.1371/journal.pcbi.1012330 (PMC11376571; doi:10.1371/journal.pcbi.1012330)
Supplement: S5 Table — We performed a PCA analysis on a subset of the dataset and obtained the reported percentage of explained variances (see details in main text and Fig 3). N corresponds to the number of embryos in each set. (PDF) [file pcbi.1012330.s017.pdf]

**Dataset subset**	$\boldsymbol{N}$	**Comp. 1 (%)**	**Comp. 2 (%)**	**Comp. 3 (%)**
Whole dataset	1618	70.63	19.16	5.83
Non treated (all temperatures and strains)	129	72.90	14.50	6.39
Treated (all temperatures and strains, RNAi or mutant)	1308	70.69	19.26	5.81
Non treated at 18°C (all strains)	58	52.03	28.31	10.61
Non treated at 23°C (all strains)	71	71.48	15.31	6.61
Depleting kinetochore-related proteins (group kt)	86	57.25	28.67	5.55
