# Supplementary material for: Unveiling inter-embryo variability in spindle length over time: Towards quantitative phenotype analysis
Source: PLoS Comput Biol. 2024 Sep 5;20(9):e1012330. doi: 10.1371/journal.pcbi.1012330 (PMC11376571; doi:10.1371/journal.pcbi.1012330)
Supplement: S6 Table — The listed treatments performed at 18°C were achieved by RNAi on the TH27 strain and compared to the corresponding L4440 treated embryos. In contrast, air-2 was a mutant reported as temperature sensitive, although we already observed phenotype at permissive temperature. It was compared to non-treated embryos from the TH27 strain at the closest temperature. Distributions are represented at S3 Fig. (PDF) [file pcbi.1012330.s018.pdf]

**Gene / Target**	p **component 1**	p **component 2**	p **component 3**
tpxl-1(RNAi) 18°C	1.7×10^{-9}	0.50	3.3×10^{-9}
cls-2(RNAi) 18°C	0.059	4.6×10^{-4}	9.3×10^{-8}
klp-19(RNAi) 18°C	0.48	0.20.	0.0046
air-2(or207ts) 15°C	0.0039	3.5×10^{-10}	5.7×10^{-5}
