# Supplementary material for: Unveiling inter-embryo variability in spindle length over time: Towards quantitative phenotype analysis
Source: PLoS Comput Biol. 2024 Sep 5;20(9):e1012330. doi: 10.1371/journal.pcbi.1012330 (PMC11376571; doi:10.1371/journal.pcbi.1012330)
Supplement: S9 Table — We trained a logistic regression with wormbase-known and -predicted interactors (column known interaction to true) among tested proteins in our dataset and predicted additional interactors marked as True in column predicted interaction (§5 in S1 Methods). (PDF) [file pcbi.1012330.s021.pdf]

Gene / Target	Coef. 1	Coef. 2	Coef. 3	Known interaction	Predicted interaction
air1	-4.21	1.17	-9.40	True	True
cdk1	-1.67	-5.07	-13.80	False	True
tpxl1-18C	-8.24	-3.02	-20.48	False	True
