# Supplementary material for: Unveiling inter-embryo variability in spindle length over time: Towards quantitative phenotype analysis
Source: PLoS Comput Biol. 2024 Sep 5;20(9):e1012330. doi: 10.1371/journal.pcbi.1012330 (PMC11376571; doi:10.1371/journal.pcbi.1012330)
Supplement: S10 Table — We trained a logistic regression with wormbase-known and -predicted interactors (column known interaction to true) among tested proteins in our dataset and predicted additional interactors marked as True in column predicted interaction (§5 in S1 Methods). (PDF) [file pcbi.1012330.s022.pdf]

Gene / Target	Coef. 1	Coef. 2	Coef. 3	Known interaction	Predicted interaction
JEP5mbk2	-0.75	-0.44	-5.19	False	True
air1	-4.21	1.17	-9.40	True	True
cdk1	-1.67	-5.07	-13.80	False	True
clip1-18C	-0.92	1.17	-6.73	False	True
cls2-18C	-1.12	3.49	-16.64	False	True
dyci1	-1.00	-8.62	-4.65	False	True
dyci1ebp2	-1.76	-6.55	-2.07	False	True
dyrb1	-1.13	-4.03	-3.28	False	True
gpb1	0.59	-5.90	-11.00	False	True
klp16	-0.36	-2.08	-2.71	False	True
klp19	-0.60	-3.93	-4.95	False	True
klp7	-0.82	-0.34	-6.47	False	True
mbk2	-1.19	-3.64	-0.41	False	True
par4	-0.37	-1.02	-5.19	False	True
plk1-18C	0.42	-8.71	-6.00	True	True
spd2-18C	-2.07	-1.28	-5.67	False	True
spn4	0.53	-2.64	-4.55	False	True
such1L4440	0.42	-6.65	-7.32	False	True
such1dylt1	0.52	-6.24	-8.41	False	True
such1mdf1	0.62	-5.82	-3.12	False	True
such1mdf1L4440	0.69	-6.70	-3.81	False	True
such1mdf1dylt1	0.78	-6.78	-2.22	False	True
tpxl1-18C	-8.24	-3.02	-20.48	False	True
unc59	-0.18	1.24	-10.09	False	True
zyg11	-0.77	-9.89	-0.44	False	True
