# Supplementary material for: Unveiling inter-embryo variability in spindle length over time: Towards quantitative phenotype analysis
Source: PLoS Comput Biol. 2024 Sep 5;20(9):e1012330. doi: 10.1371/journal.pcbi.1012330 (PMC11376571; doi:10.1371/journal.pcbi.1012330)
Supplement: S11 Table — Mann-Whitney test comparing coefficients of genes for which a significative difference (p < 0.01) was found for all coefficients. The listed treatments were achieved by RNAi on the TH27 strain and compared to the L4440-treated control embryos at the same temperature. (PDF) [file pcbi.1012330.s023.pdf]

**Gene / Target**	**Temperature**	p **component 1**	p **component 2**	p **component 3**
let99-23C	23°C	1.70559e-05	9.44314e-07	0.000483833
ani2-23C	23°C	8.91965e-07	0.000708873	7.99249e-08
lin5-18C	18°C	9.59369e-09	3.17415e-07	8.97462e-05
gpa16	18°C	2.20445e-05	8.2908e-05	0.000472105
zen4	18°C	4.72773e-05	5.74555e-09	3.43699e-05
cdk1	18°C	3.58523e-06	0.000695534	0.00184639
spd1	18°C	0.00854128	5.45828e-08	0.000113455
spd2-18C	18°C	2.333e-09	0.00623252	1.9433e-05
