# Supplementary material for: Unveiling inter-embryo variability in spindle length over time: Towards quantitative phenotype analysis
Source: PLoS Comput Biol. 2024 Sep 5;20(9):e1012330. doi: 10.1371/journal.pcbi.1012330 (PMC11376571; doi:10.1371/journal.pcbi.1012330)
Supplement: Attachment — Submitted filename: Cover_letter_and_point_by_point_s.pdf [file pcbi.1012330.s024.pdf]

igdr

UMR 6290 CNRS – UR1
Faculté de Médecine
2 avenue du Professeur Léon Bernard
CS 34317, 35043 Rennes cedex, France
jacques.pecreaux@univ-rennes1.fr
T. +33 (0)2 23 23 45 03
F. +33 (0)2 23 23 44 78
helene.bouvrais@univ-rennes1.fr
T. +33 (0)2 23 23 40 08

Rennes, 14th June 2024

Prof. Changbong Hyeon, PhD, academic editor,
Prof. Jason Haugh, PhD, section editor,
PLOS Computational Biology

Dear editors,

We are pleased to submit our revised manuscript entitled "Unveiling Inter-Embryo Variability in Spindle Length over Time: towards Quantitative Phenotype Analysis." by Yann Le Cunff *et al.* to PLOS Computational Biology (reference PCOMPBIOL-D-24-00330).

Beyond the details of the changes provided in the point-by-point below, we have rewritten the discussion to detail and highlight what we learned about the spindle by classifying the variation of its time-elongation curve with PCA without a priori compared to previous approaches. We also discuss in further detail the implications of our findings regarding the understanding of the spindle mechanisms, especially for the unexpected spindle-shortening archetype accounting for variability in all conditions, including non-treated. We have, however, limited ourselves to discussion since it is out of the scope of this paper to perform the experiments to test these findings thoroughly. The second major change lies in adding a logistic regression on the three PCA coefficients to exploit the predictive power of our method. By doing so, we find genes involved in the same mechanisms, termed interactors in a wide meaning. We added three examples of its use at the end of the results section, to demonstrate the added value compared to known interactors available in large databases. The corresponding code was added to the git repository referenced in the methods to be re-usable by others. We are deeply grateful to both referees for their insightful comments and suggestions. Their feedback has significantly improved our manuscript, enhancing both the biological significance of our findings and the re-usability of our approach by the scientific community. We believe that the manuscript is now ready for publication and can provide any additional information upon request.

Yours sincerely,

Jacques Pécréaux

Reviewer #1:

The authors of this interesting and useful paper approach the problem of dynamic variability of mitotic spindle using data science approach, without fitting or modeling or pre-determining quantitative features of the spindle. They take measured time series of spindle lengths in metaphase and anaphase under many control and perturbed conditions, and apply PCA analysis to these time series. Most of variability is captured by just three first eigenvectors, which the authors call archetypes. Those archetypes have intuitive interpretation: one of them characterizes the average spindle length, another - the elongation rate, and the third one - transient 'weakening' of the spindle in late metaphase. By choosing various subsets of the data, the authors demonstrate that these archetypes are robust. They use ML to show that weights of the archetypes at the anaphase onset predict the anaphase elongation rate. The paper is sound and timely.

We are grateful to the reviewer for his very positive comment and do our best to improve our manuscript following her/his comments.

I have the following three general comments:

1) It is a bit unsatisfying that there is little insight into molecular mechanisms of mitosis. After all, many specific perturbations are used. I sense that by looking at the weights of the archetypes in different conditions we can learn more about plausibility of many mechanistic models of the spindle dynamics proposed in the last decade.

We added a last section to the results , entitled "the PCA's three coefficients are a predictive tool", to exemplify how PCA coefficients can be used to predict gene interactions and mentioned it at the end of the abstract.

2) Aside from criticism of studies [2,23], the features pre-selected there were very useful for classifying mitotic scenarios. Are there any quantitative correlations between the weights of the archetypes and these previously introduced signatures of the spindle elongation. Such analysis would be enlightening.

We apologise for the lack of clarity in our manuscript about this comparison. This comparison mentioned in the abstract, "The two first archetypes were consistent with standard measures in the field…" has been clarified.

We put the related paragraph of the results section into a dedicated subsection entitled "comparing with existing spindle characteristics" to improve readability. We rewrote it to clarify the importance of this previous study and what our method is bringing. We also better highlighted the improvements we claim from our method.

We also rewrote the beginning of the discussion to highlight the links between our approach and Farhadifar ones.

3) Writing is mostly clear, but overly 'wordy'. I felt, when reading, that the main text could be significantly shortened - there are many repetitions and meandering descriptions.

We rewrote the manuscript to better separate the results and discussion and remove repetitions to be more focused on the point. However, we left some explanations likely obvious to experts in the field to keep our paper accessible to a broad audience. We indeed intended it for both bioinformaticians and cell biologists.

Also, the section on using ML to show that weights of the archetypes at the anaphase onset predict the anaphase elongation rate probably belongs to the end of the results - it breaks the flow, and the robustness results have to be shown before it.

We moved the section "Anaphase onset as a turning point" after the part "Robustness of the main archetypes," as nicely suggested. However, we opted not to put it at the end of the results but before the section "From archetypes to phenotypes," as this latter offers an applicative opening to our method. We also better motivated this section to highlight how it helps understand the biological implications of our results using PCA.

Reviewer #2:

The manuscript by Le Cunff et al. tackles a long-standing issue in modeling and understanding the in vivo behavior of biological structures – the variability that occurs among different measurements of the same parameter. The authors utilized PCA instead of a more traditional mathematical model approach with the aim of better accounting for variability in raw data. PCA analysis allowed the authors to "extract" different types of spindle elongation behaviors from multiple experimental manipulations of the first mitosis in C. elegans embryos and demonstrate that these "archetypes" are present in unperturbed embryos. This work provides solid support for relying less on fitting models to data based on a priori knowledge and instead allowing unbiased methods to highlight features that may not be appreciated or identified when using a pre-existing framework. This proof of principle may be useful to multiple fields of biology.

From a cell biologist's perspective, it would be beneficial to dive a little deeper into how this approach our understanding of spindle elongation.

We rewrote the first half of the discussion to better highlight how our approach advanced our understanding of the spindle.

While the diverse range of spindle-associated proteins subjected to partial RNAi is commendable, much of the analysis was primarily focused on seeing how the archetypes derived from PCA were affected by different subsets of the experimental data. Further analysis of specific RNAi conditions that were not highlighted in the main text may yield new insights on characterized spindle proteins being involved in more pathways than previously known.

We re-organised the manuscript to better highlight the interest of our approach, restricting the establishment of the method to results sections 3.1 and 3.2. We also developed the logistic regression as a predictive tool and demonstrated it on examples (cf. subsection "The PCA's three coefficients are a predictive tool"). Finally, we also rewrote the first half of the discussion to clarify the biological implications of our work. Having said that, it is however not in the scope of

this paper to use this technique in a real biological project with solid outcomes based on a set of experiments beyond the bioinformatics predictions.

Furthermore, the authors propose that the RNAi and PCA approach shown here may help to identify novel spindle proteins that were previously elusive due to hard-to-interpret phenotypes; a concrete example of this case (i.e. showing a protein depletion that the PCA matches to a specific archetype and verifying a new function for that protein) would significantly boost the impact of the work.

We added a last section to the results entitled "The PCA's three coefficients are a predictive tool" to exemplify how PCA coefficients can be used to predict interactions and mentioned it at the end of the abstract.

Nevertheless, the general premise of the work seems sound and logical; the experiments and computations appear rigorous and well-designed and this work seems suitable for publication in PLOS Computational Biology.

We are very thankful to the reviewer for his/her support to our work.

Major points

1. A stronger emphasis on how PCA differs from previous mathematical a priori modeling of spindle dynamics would be helpful.

We edited the introduction to better motivate the grounds for making PCA. As numerous studies already investigated the spindle length, we rewrote the first half of the discussion to present the novel insights brought by our approach in comparison to previous studies.

In our view, the ability to predict gene interactions based on PCA coefficients is also very useful to support experimental investigations of the spindle. We thus pushed forward the method along that line and provided three examples.

The motivation for using PCA instead of relying on well-established models in the field of spindle dynamics could be better described in the introduction.

In the introduction, when reviewing the state of the art, at the end of the paragraph, we better motivate how our approach contrasts with these existing ones. At the end of the paragraph "Diversity of spindle elongation phenotypes", we highlight how PCA is original to cope with this diversity. We also added a paragraph at the beginning of the section "Using projection methods to obtain a model-free description of variability" to highlight better the rationale of the technical choices done in our approach and how it is discriminative from previous art.

What exactly is gained by this approach? Additional explanation of how a priori mathematical models have not fully recapitulated spindle behavior would be beneficial to demonstrating the value for an approach that can find "hidden" features.

We rewrote the discussion to better highlight the gains offered by our approach. In particular, in the third paragraph starting by "Beyond finding archetypes informative …", we discussed the implication of finding the third archetype as an "hidden" feature, present even in non-treated embryos.

2. A definitive application of this approach to identify a novel spindle-related function of a previously ambiguous protein would be extremely convincing of the future application of this PCA approach.

We are thankful to the reviewer for highlighting the ability of our approach to offer prospective knowledge. We added a subsection entitled "the PCA's three coefficients are a predictive tool" at the end of the result section to highlight three examples of the use of the PCA coefficient map to sort out various opened questions about spindle-related functions. We used debated cases, with however some experimental results recently published, to decide the value of our method predictions.

Minor points

1. The provided supplemental data and tables (e.g. Table S6) highlight that certain RNAi conditions contribute strongly to one archetype. It would be intriguing if any of the RNAi conditions tested in the dataset have an almost equal contribution across all three archetypes, suggesting roles in multiple aspects of the mitotic spindle that may not have been previously suggested/identified. There are multiple proteins listed in the supplement (i.e. kinesins, TAC-1, ZYG-9) that might be predicted to have multiple effects on spindle elongation.

We are thankful to the reviewer for this interesting suggestion. We performed the test and reported it in the table S11. The interpretation of the results appears however beyond the scope of this paper.

2. This study and cited papers (55,56), use computational calculations of centrosome position, how does this lead to "10 nm accuracy", beyond the resolution limit of light microscopy?

In the paragraph "Centrosome-tracking assay, filtering and getting spindle length" of the method, we briefly explained the ground of the method and the measuring of accuracy. We set to keep it brief as it was already published.

3. Figure 5A is visually difficult to interpret. Having so many lines plotted together across a gradient of color and textures makes it difficult to discern which line corresponds to what condition and KLP-7 intensity.

We kept only the average on Fig 5A and offer supplemental figure S8 with the detail on individual embryos, with one panel per condition for clarity.

4. Throughout the manuscript requires editing for clarity/accuracy.

We strongly edited the manuscript, also in an attempt to clarify the text.
